# Editorial: Role of nutrition in skeletal muscle atrophy and sarcopenia

**DOI:** 10.3389/fnut.2024.1395491

**Published:** 2024-05-03

**Authors:** Tao Tong, Helong Quan, Chang Keun Kim, Weicai Zeng

**Affiliations:** ^1^Key Laboratory of Precision Nutrition and Food Quality, Key Laboratory of Functional Dairy, Ministry of Education, College of Food Science and Nutritional Engineering, China Agricultural University, Beijing, China; ^2^Key Laboratory of Safety Assessment of Genetically Modified Organism (Food Safety), Ministry of Agriculture, Beijing, China; ^3^Beijing Laboratory for Food Quality and Safety, Beijing, China; ^4^School of Sports Science and Physical Education, Research Center of Sports and Health Science, Northeast Normal University, Changchun, China; ^5^Department of Human Physiology, Korea National Sport University, Seoul, Republic of Korea; ^6^Antioxidant Polyphenols Team, Department of Food Engineering, Sichuan University, Chengdu, China

**Keywords:** skeletal muscle atrophy, sarcopenia, protein synthesis/proteolysis, nutrition, dietary intervention

Approximately 40%–50% of the body mass consists of skeletal muscle, which is important for human energy metabolism. Its quality and functional integrity are crucial to maintaining the normal function and metabolic homeostasis of the musculoskeletal system (1). Muscle atrophy reduces strength and endurance, which restricts activity, decreases the quality of life, increases the risk of falls and fractures, and starts a vicious cycle of muscle disuse that exacerbates muscle weakness and loss. The atrophy of skeletal muscle has various causes, including aging, cancer, metabolic diseases, sepsis, bed rest, and denervation (2). Sarcopenia, known as the most prevalent type of skeletal muscle atrophy in humans, is primarily associated with aging. Notably, as the aging of the global population continues to deepen, sarcopenia has become a serious public health problem, causing huge direct or indirect socioeconomic burdens (3). Limited success has been achieved in clinical trials testing therapeutics against skeletal muscle atrophy and sarcopenia (4). Therefore, finding dietary approaches for the prevention and treatment of muscle atrophy and sarcopenia has been a research hotspot in the fields of nutrition, medicine, and biology.

Highly dynamic, skeletal muscle reacts to a variety of stimuli, particularly mechanical load variations. The main cause of muscle atrophy in healthy individuals is muscular disuse, which also impacts protein expression, metabolism, skeletal muscle phenotype, and morphological features (5). Decreased physical activity is mostly due to reduced ambulatory activity, lockdowns imposed, illnesses necessitating bed rest, or discomfort or impairments from chronic conditions. Spinal cord injury is a severe, incapacitating condition that causes skeletal muscle innervation loss, lower motor function, and considerably reduced skeletal muscle load, leading to atrophy (6). Xu et al. indicated that oxidative stress injury and inflammation represented the primary mechanisms of skeletal muscle atrophy following spinal cord injury. This included various pathways, including the autophagic lysosome system, the ubiquitin-proteasome system, the IGF-1/PI3K/Akt/mTOR signaling pathway, and the hypothalamus-growth hormone-IGF-1 axis (Xu et al.).

Inflammation is essential to the pathology of illnesses linked to dysfunctional skeletal muscles (7). A number of illnesses, such as chronic obstructive pulmonary disorder (COPD) and asthma, are marked by persistent inflammation or an increase in inflammatory mediators. Even though the pathologies of these disease states vary, they are all characterized by skeletal muscle mass loss and physiological disruption. Pro-inflammatory cytokines play a major role in the chronic inflammation that characterizes several of these diseases (8). The data from 3,389 participants in the National Health and Nutrition Examination Survey (NHANES) from 1999 to 2006 and 2011 to 2018 showed a significant positive association between the risk of sarcopenia in asthmatic patients and their dietary inflammatory index levels, as reported by Lin et al. Therefore, decreasing the intake of these pro-inflammatory elements may help prevent sarcopenia. To understand the function of nutrition in patients with coexisting COPD and sarcopenia, Nan et al. summarized the intrinsic mechanisms of both conditions.

Diet is an important determinant of skeletal muscle atrophy. Muscle atrophy can be caused by a variety of dietary variables, including insufficient protein consumption, vitamin D deficiency, and a diet low in antioxidants and long-chain polyunsaturated fatty acids (9). After screening data selected from the 2011–2014 NHANES, including 6,019 American adults, Wu et al. showed that the composite dietary antioxidant index and the dietary antioxidants zinc, vitamin E, and selenium were significantly correlated with handgrip strength in males, while only zinc was related to handgrip strength among dietary antioxidants in females. Additionally, eating a diverse range of foods can lower the risk of unfavorable health outcomes like physical dysfunction and death. Dietary diversity score refers to the number of various foods or food groups within a specific timeframe, where higher scores signify a broader range of foods consumed. Using the data from 6,624 older Chinese adults, Du et al. showed that sarcopenia risk was lower in those with higher dietary diversity scores for the total diet, animal-based diet, and plant-based diet. Therefore, consuming a diverse range of foods is crucial for maintaining bodily function (Du et al.).

In summary, this Research Topic provides the most recent information on the impact of diets or dietary components on skeletal muscle atrophy and sarcopenia while updating the molecular mechanisms that mediate and orchestrate muscle mass loss in disease. It offers new insights into the possible strategies for the management and prevention of these conditions. Meanwhile, due to the complex pathogenesis of skeletal muscle atrophy and existing inter-individual variability, precise nutrition for individuals with skeletal muscle atrophy will be a future research trend.

## Author contributions

TT: Writing – original draft, Writing – review & editing. HQ: Writing – original draft, Writing – review & editing. CK: Writing – original draft, Writing – review & editing. WZ: Writing – original draft, Writing – review & editing.
